# miR-29a and miR-15b Modulate SARS-CoV-2 Beta and Omicron Infection in Human Lung Epithelial Cells

**DOI:** 10.3390/ijms27135847

**Published:** 2026-06-29

**Authors:** Elena Criscuolo, Nicola Mosca, Benedetta Giuliani, Matteo Castelli, Armando Di Palo, Mariaceleste Pezzullo, Roberto Burioni, Aniello Russo, Nicola Clementi, Nicoletta Potenza

**Affiliations:** 1 Laboratory of Microbiology and Virology, Vita-Salute San Raffaele University, 20132 Milan, Italy; criscuolo.elena@hsr.it (E.C.); giuliani.benedetta@hsr.it (B.G.); castelli.matteo@hsr.it (M.C.); burioni.roberto@hsr.it (R.B.); 2 Department of Environmental, Biological and Pharmaceutical Sciences and Technologies, University of Campania «L. Vanvitelli», 81100 Caserta, Italy; nicola.mosca@unicampania.it (N.M.); armando.dipalo@genomix4life.com (A.D.P.); mariaceleste.pezzullo@unicampania.it (M.P.); aniello.russo@unicampania.it (A.R.); 3 Laboratory of Microbiology and Virology, IRCCS-San Raffaele Scientific Institute, 20132 Milan, Italy

**Keywords:** SARS-CoV-2, microRNA, miR-29a, miR-15b, Beta variant, Omicron variant, viral replication, lung epithelial cells

## Abstract

Host microRNAs (miRNAs) are widely proposed as innate antiviral effectors against SARS-CoV-2, yet whether they actually restrict infection in lung epithelial cells remains unresolved. Two of the most-cited candidates, miR-29a-3p and miR-15b-5p, are predicted to bind both the viral genome and key entry/trafficking factors such as Furin and ATG9A, but functional evidence is fragmented and often contradictory. Here, we put both miRNAs to the test in human Calu-3 cells infected with the SARS-CoV-2 Beta and Omicron BA.1 variants, using parallel gain- and loss-of-function strategies coupled to RT-qPCR of viral and cellular transcripts and back-titration of infectious progeny on VeroE6/TMPRSS2 cells. Both miRNAs transiently suppressed viral gene expression at 6 hpi, but this early dampening was followed by a marked transcript rebound at 24 hpi, especially for Omicron, with virtually no impact on total extracellular viral RNA. More strikingly, miR-15b modulation enhanced infectious virus output during Beta infection, and miR-29a overexpression boosted Omicron BA.1 infectivity, while Furin, ATG9A, AKT3, and TFEB showed only modest, condition-dependent shifts. Rather than acting as clean antiviral effectors, miR-29a and miR-15b emerge as context-dependent modulators that can paradoxically favor SARS-CoV-2 replication—a cautionary signal for miRNA-based antiviral strategies.

## 1. Introduction

Since the emergence of severe acute respiratory syndrome coronavirus 2 (SARS-CoV-2) in late 2019, the COVID-19 pandemic has highlighted the need to understand virus–host interactions at a molecular level to identify new antiviral targets. Beyond viral proteins and canonical immune pathways, host post-transcriptional regulators such as microRNAs (miRNAs) have attracted growing attention as potential modulators of coronavirus infection and disease outcome [1]. miRNAs are small non-coding RNAs that fine-tune gene expression by binding complementary sequences in viral and cellular RNAs, thereby shaping innate immunity, cell survival, and metabolic adaptations during infection [2,3].

Several in silico and experimental studies have mapped a complex miRNA–SARS-CoV-2 interactome, revealing multiple predicted binding sites for host miRNAs along the viral genome and showing that infection can remodel cellular miRNA expression profiles [4]. Our previous work was the first to experimentally validate five human miRNAs binding to viral sequences through an unbiased screening based on filtering criteria applied to bioinformatic analyses and cell-based reporter systems [5]. Among those miRNAs, hsa-miR-29a-3p (miR-29a) and hsa-miR-15b-5p (miR-15b) were further validated by other studies through different approaches [4]. Members of the miR-15/16 family and the miR-29 family are of particular interest, as they have been implicated in interferon signaling, T-cell differentiation, autophagy, and stress responses, and are predicted to bind both viral RNAs and key host factors, including the protease Furin and the autophagy regulator ATG9A [6,7,8]. In this context, miR-29a and miR-15b have been proposed as candidate antiviral miRNAs capable of directly targeting SARS-CoV-2 RNA and indirectly modulating pathways required for efficient replication [4].

However, the functional evidence for an antiviral versus pro-viral role of these miRNAs remains controversial. Some reports suggest that overexpression of selected miRNAs can silence viral sequences or reduce Spike (S) expression in reporter systems, supporting an antiviral model [9]. Other studies, as well as emerging data on SARS-CoV-2-induced miRNA deregulation, point to more nuanced and sometimes opposing effects, in which the same miRNA may restrict early steps of infection but facilitate later stages of the viral life cycle or immune evasion [10,11]. This divergence underscores a broader debate in the field on whether miRNAs should be viewed primarily as innate antiviral effectors or as host factors that viruses co-opt for their own replication.

The present work addresses this gap by functionally dissecting the role of miR-29a and miR-15b during SARS-CoV-2 Beta and Omicron BA.1 infection in human lung epithelial Calu-3 cells, which, despite their immortalized nature, represent a robust model for the lower respiratory tract due to their endogenous expression of ACE2 and TMPRSS2, supporting the native viral entry pathway [12,13,14,15]. By combining gain- and loss-of-function approaches with quantitative analysis of viral gene expression, infectious progeny production, and selected cellular targets, the study aims to clarify whether these miRNAs exert net antiviral or pro-viral effects under variant-specific conditions. The main conclusion is that neither miR-29a nor miR-15b behaves as a straightforward antiviral factor; instead, their manipulation can paradoxically enhance viral progeny production, highlighting the complexity of miRNA–virus–host networks and cautioning against simplistic miRNA-based therapeutic strategies.

## 2. Results

### 2.1. Binding Sites for miR-29a and miR-15b on the SARS-CoV-2 Genome

**Figure 1:**
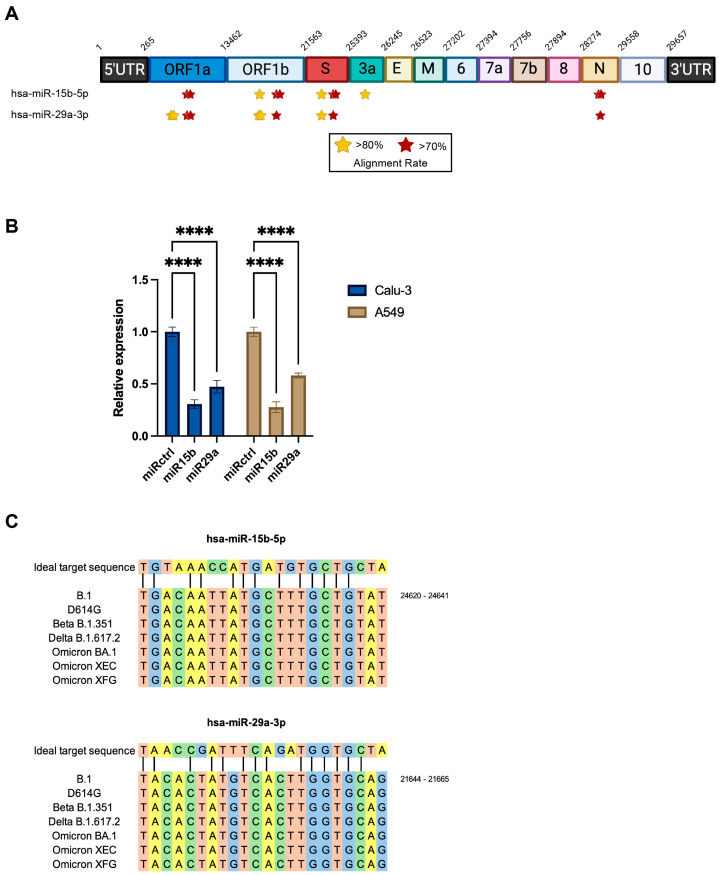
Predicted miRNA binding sites and modulation of viral RNA levels. (**A**) Schematic representation of predicted binding sites for hsa-miR-29a-3p and hsa-miR-15b-5p along the SARS-CoV-2 genome (B.1, Wuhan-Hu-1 reference, NC_045512.2), identified using miRanda (Version 3.3a), RNAhybrid 2.2 and miRDB tools. Key target regions are indicated by yellow or red stars depending on identity scores. (**B**) Quantification of S mRNA in Calu-3 and A549 cells co-transfected with S-expressing plasmid (pSpike, Wuhan-Hu-1 derived) and either control miRNA (miR-ctrl), miR-29a mimic (50 nM), or miR-15b mimic (50 nM), measured by RT-qPCR at 48 h post-transfection. Data are normalized to GAPDH housekeeping gene; bars represent mean ± SD of triplicate biological replicates from at least two independent experiments. Mean ± SD, **** *p* < 0.0001. (**C**) Alignment of the two conserved target sequences recognized by hsa-miR-29a-3p and hsa-miR-15b-5p in the S coding sequence of Wuhan B.1, D614G, Beta B.1.351, Delta B.2.617.2, Omicron BA.1, XEC, and XFG VOCs.

In silico prediction identified multiple putative binding sites for miR-15b-5p and miR-29a-3p along the SARS-CoV-2 genome, distributed within ORF1a, ORF1b, Spike (S), ORF3a, and the Nucleocapsid (N) region, with several sites displaying alignment rates above 80% (Figure 1A, Tables S1 and S2). These findings suggest that both miRNAs may directly interact with viral RNA segments involved in replication and structural protein expression. As stated in the Introduction, some of those interactions were experimentally validated [4,5]. Given the presence of multiple conserved binding sites for miR-29a and miR-15b across SARS-CoV-2 ORF1a, ORF1b, S, and N genes, these targets were selected for investigation owing to their pivotal roles in viral replication (ORF1a/b encode non-structural proteins, including RdRp) and virion assembly/transmission (S and N as structural proteins). Experimental validation, however, prioritized S and N genes, as the S protein facilitates host cell entry and elicits strong immunogenicity conducive to reporter-based assays, whereas N is highly abundant and stabilizes the viral genome for straightforward detection. The availability of a plasmid overexpressing the natural and ancestral S protein from the B.1 strain allows us to test the miRNA’s ability to inhibit its expression in two different human lung adenocarcinoma epithelial cells in the absence of other viral genes to avoid confounding effects. Indeed, miR-15b and miR-29a led to a marked reduction (*p* < 0.0001) in plasmid-driven Spike expression, with miR-15b exerting the strongest inhibitory effect in both cell lines at 48 h post-transfection, a time point selected to allow sufficient plasmid-driven S expression and subsequent miRNA-mediated repression to become detectable (Figure 1B) [16]. These results provided initial evidence that both miRNAs can recognize and suppress S gene expression, although they may not indicate an antiviral effect in the context of infection, when a complex interplay between virus and host occurs. Notably, sequence alignment among different S variants of concern (VOCs) revealed high conservation of the predicted target sites (Figure 1C), suggesting a significant role in viral physiology. Based on these findings, we selected the Beta and Omicron variants for further analyses and Calu-3 as an in vitro model of lung epithelial infection.

### 2.2. Set Up of the Experimental System

To investigate the impact of SARS-CoV-2 infection on miRNA expression, we employed human lung epithelial Calu-3 cells, a well-established in vitro model for respiratory viral infections due to their physiological relevance and high permissiveness to SARS-CoV-2. Cells were infected with the Beta (B.1.351) and Omicron BA.1 (B.1.1.529) variants, selected as prototype SARS-CoV-2 lineages because they exemplify distinct biological and epidemiological trajectories. Beta is characterized by a spike mutational pattern that confers marked escape from first-generation vaccine-elicited and convalescent neutralizing antibodies, but only modest gains in transmissibility compared with ancestral lineages. In contrast, Omicron BA.1 combines extensive spike diversification with pronounced immune evasion, a shift in cell entry pathways and tissue tropism, and substantially higher intrinsic transmissibility, enabling rapid global dominance even in highly vaccinated or previously exposed populations.

**Figure 2:**
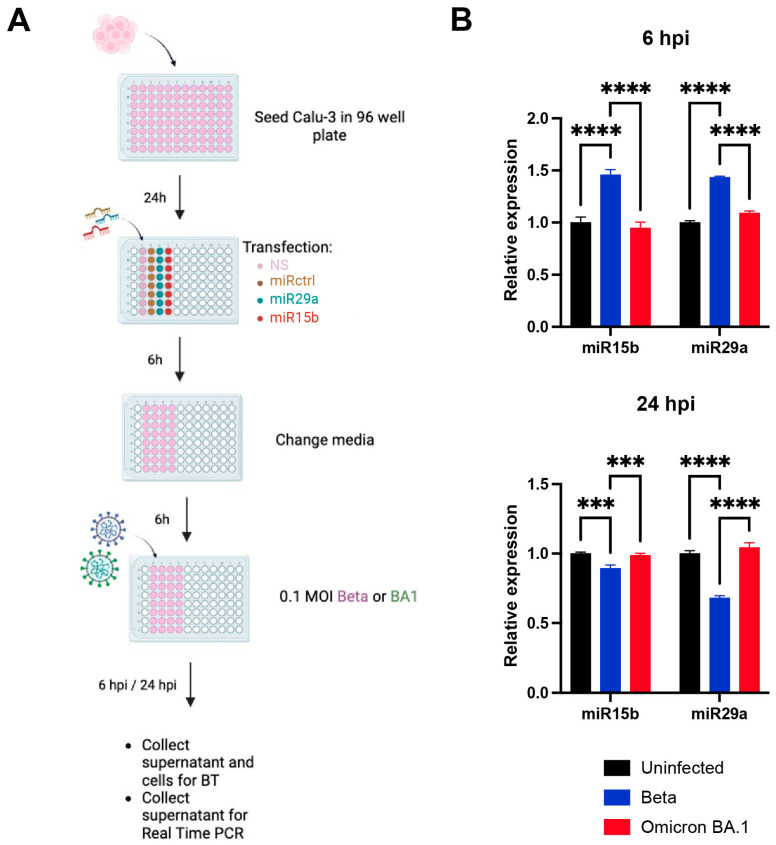
Experimental system setup. (**A**) Schematic representation of the experimental workflow designed to assess both the effects of SARS-CoV-2 infection on miRNA expression and the influence of miRNAs on viral infection. (**B**) Endogenous miR-15b and miR-29a levels were evaluated in non-transfected infected (Beta or Omicron BA.1) and matched uninfected Calu-3 cells at 6 and 24 hpi. Each variant was tested in an independent experimental session with its own uninfected control. Data are normalized to RNU6B reference transcript. Each replicate represents an independently seeded and infected well. Bars represent mean ± SD of *n* = 3 biological replicates. Statistical significance was assessed by unpaired two-tailed Student’s *t*-test. **** *p* < 0.0001, *** *p* < 0.001.

To modulate the experimental targets, cells were transfected with the miRNAs of interest 12 h prior to infection (Figure 2A). Transfection was performed 12 h before viral inoculation, followed by a complete medium change, to ensure full RISC loading of the transfected miRNAs and to minimize confounding effects related to residual transfection reagents, transient membrane permeability changes, or cellular stress responses that could interfere with viral entry or downstream analyses. Following infection, the expression levels of the miRNAs of interest were then quantified to assess whether viral replication affects intracellular miRNA profiles (Figure 2B). These results were interpreted in the context of recent literature, which reports heterogeneous findings: some studies describe no significant changes in specific miRNA levels upon SARS-CoV-2 infection, whereas others report marked modulation, suggesting that miRNA regulation may depend on the viral variant, time post-infection, and the cellular model employed [17,18,19]. Our results indicated that only the Beta variant markedly impacted both miR-15b and miR-29a levels, significantly increasing their expression at 6 h post-infection (hpi) (*p* < 0.0001), followed by a significant decrease at 24 hpi (*p* < 0.0001).

### 2.3. Effect of miR-29a and miR-15b Modulation on Beta-Infected Cells

#### 2.3.1. miRNA Mimic Experiments

**Figure 3:**
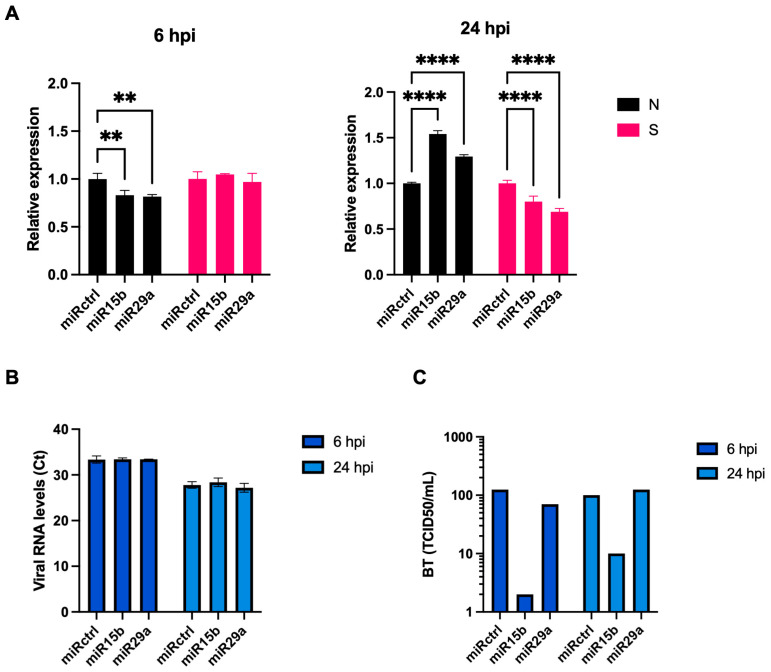
Effect of miR-29a and miR-15b modulation on Beta-infected cells. (**A**) N (nucleocapsid) and S (Spike) gene expression at 6 and 24 h post-infection (hpi) in Calu-3 cells infected with SARS-CoV-2 Beta variant (0.1 MOI) and previously transfected with control miRNA, miR-29a mimic, or miR-15b mimic (50 nM). Expression was measured by RT-qPCR and expressed as fold change relative to control-miRNA-transfected cells at each time point. (**B**) Viral RNA levels in cell culture supernatants at 6 and 24 hpi, quantified by RT-qPCR targeting the nucleocapsid (N) RNA and presented as Ct (cycle threshold) values. (**C**) Infectious viral progeny in supernatants from mimic-transfected Beta-infected cells collected at 6 and 24 hpi, determined by back titration on VeroE6/TMPRSS2 cells using serial dilutions (10^−1^ to 10^−8^) and expressed as TCID_50_/mL. For panels (**A**,**B**), two independently seeded and transfected biological wells per condition were each assayed in duplicate by RT-qPCR (*n* = 4); for panel (**C**), each value represents a single determination from triplicate infection wells per dilution. Data are from a single experimental session conducted under BSL-3 containment. Bars represent mean ± SD. ** *p* < 0.01; **** *p* < 0.0001.

When Calu-3 cells were pre-transfected with either miR-29a or miR-15b mimics and subsequently infected with the SARS-CoV-2 Beta variant (0.1 MOI), viral N gene expression was assessed as a predicted target for both, and the results showed a significant reduction at 6 hpi in both conditions (*p* < 0.01, Figure 3A). By 24 hpi, however, N expression rebounded and exceeded control levels in both mimic-transfected cells (*p* < 0.0001). S gene expression was assessed as well, as the in silico analysis predicted even more binding sites on its sequence, and the results showed that it was not affected at 6 hpi, while a pronounced reduction was observed at 24 hpi in both cases (*p* < 0.0001). The N and S transcripts were quantified using independent, gene-specific primer sets; their divergent temporal profiles are consistent with the known differential accumulation kinetics of SARS-CoV-2 subgenomic RNAs [20,21], rather than reflecting assay cross-reactivity. To determine if these intracellular dynamics translated into altered viral egress, we quantified viral RNA in the cell culture supernatants, which specifically reflects the genomic RNA packaged into released virions. Total extracellular viral RNA in cell supernatants, measured by RT-qPCR targeting the N region, confirmed productive infection with no significant differences between mimic treatments and control at both time points, indicating that overall viral particle production was not substantially impaired (Figure 3B). Viral RNA levels reflect genome abundance but do not discriminate between infectious and non-infectious virus particles. Critically, quantification of infectious viral particles by back titration (BT) assay revealed that miR-15b mimic-treated supernatants contained markedly reduced infectious titers compared with controls, with a ~2 log_10_ reduction at 6 hpi and a ~1 log_10_ reduction at 24 hpi, whereas miR-29a-transfected cells showed comparable titers to control at both time points (Figure 3C). This dichotomous pattern, suppressed infectivity from miR-15b despite transient reductions in viral transcript levels, coupled with enhanced infectivity from miR-29a at late infection, suggests that these miRNAs differentially modulate post-translational steps in virion assembly or release, e.g., by modulating cellular proteases (e.g., Furin) or autophagy pathways (e.g., ATG9A).

#### 2.3.2. miRNA Inhibitor Experiments

**Figure 4:**
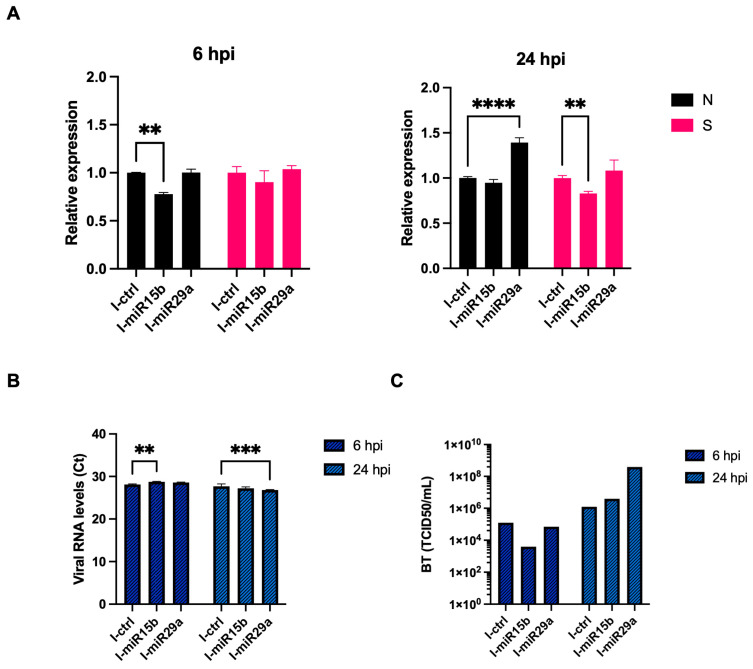
miRNA inhibitor effects in Beta-infected Calu-3 cells. (**A**) N and S gene expression at 6 and 24 hpi in Calu-3 cells infected with SARS-CoV-2 Beta variant (0.1 MOI) and transfected with the control inhibitor (I-Ctrl, 50 nM scrambled anti-miRNA), anti-miR-29a inhibitor (I-miR-29a, 50 nM), or anti-miR-15b inhibitor (I-miR-15b, 50 nM). RT-qPCR data are shown as fold change relative to the control inhibitor at each time point. (**B**) Viral RNA levels in supernatants at 6 and 24 hpi, measured by RT-qPCR. (**C**) Infectious viral titers in supernatants from inhibitor-transfected Beta-infected cells collected at 6 and 24 hpi, quantified as TCID_50_/mL by back titration on VeroE6/TMPRSS2 cells. Replicate structure is as in Figure 3. Data are from an independent experimental session from Figure 3. Bars represent mean ± SD. ** *p* < 0.01; *** *p* < 0.001; **** *p* < 0.0001.

When Calu-3 cells were pre-transfected with miR-15b or miR-29a inhibitors (I-miR-15b or I-miR-29a) and then infected with the SARS-CoV-2 Beta variant (0.1 MOI), N gene expression at 6 hpi was significantly decreased only in the presence of the miR-15b inhibitor (*p* < 0.01), while miR-29a inhibition did not appreciably change N levels compared with the inhibitor control (Figure 4A). At 24 hpi, N mRNA was higher in the miR-29a inhibitor condition than control (*p* < 0.0001), whereas I-miR-15b resulted in no change in N expression compared to control. S gene expression was similar across all conditions at 6 hpi, but at 24 hpi it was clearly reduced in cells treated with miR-15b inhibitor compared with control (*p* < 0.01). Extracellular viral RNA in supernatants showed a modest but statistically significant increase in Ct at 6 hpi upon miR-15b inhibition (~1 Ct shift, corresponding to an approximately 2-fold reduction in viral RNA; *p* < 0.01), and a decrease in Ct at 24 hpi upon miR-29a inhibition (~1.5 Ct shift, corresponding to an approximately 3-fold increase in extracellular viral RNA; *p* < 0.001), consistent with an alteration in the release of viral particles in these conditions (Figure 4B). BT assays showed that inhibition of miR-15b was associated with a modest reduction (~0.5 log_10_) in infectious titers compared with control at 6 hpi, while miR-29a inhibition yielded titers comparable to control. At 24 hpi, both inhibitor conditions produced higher infectious titers than control, with miR-29a inhibition associated with the most pronounced increase (~3 log_10_ above control), indicating a substantial enhancement of infectious particle production at the later time point (Figure 4C).

### 2.4. Effect of miR-29a and miR-15b Modulation on Omicron BA.1-Infected Cells

#### 2.4.1. miRNA Mimic Experiments

**Figure 5:**
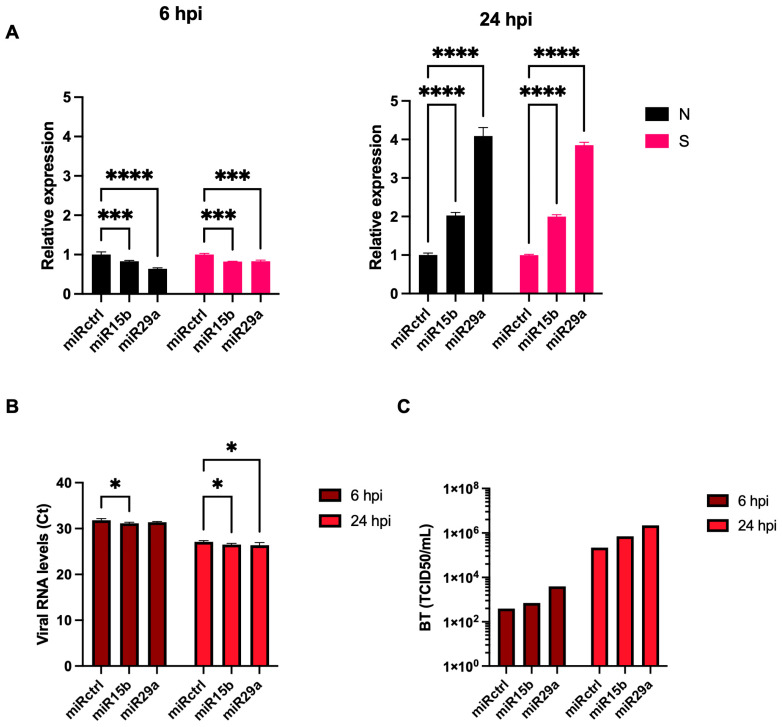
miRNA mimic effects in Omicron BA.1-infected Calu-3 cells. (**A**) N and S gene expression at 6 and 24 hpi in Calu-3 cells infected with SARS-CoV-2 Omicron BA.1 variant (0.1 MOI) and transfected with control miRNA, miR-29a mimic, or miR-15b mimic (50 nM). Expression levels were determined by RT-qPCR and are presented as fold change relative to control miRNA at each time point. (**B**) Viral RNA levels in supernatants at 6 and 24 hpi, measured by RT-qPCR. (**C**) Infectious progeny titers in supernatants from mimic-transfected Omicron-infected cells collected at 6 and 24 hpi, expressed as TCID_50_/mL based on back titration on VeroE6/TMPRSS2 cells. Replicate structure is as in Figure 3. Data are from a single experimental session conducted under BSL-3 containment. Bars represent mean ± SD. * *p* < 0.05; *** *p* < 0.001; **** *p* < 0.0001.

During Omicron BA.1 infection, miR-29a and miR-15b mimic transfection led to early reductions in N gene expression at 6 hpi similar to Beta (*p* < 0.001 and *p* < 0.0001), but the 24 hpi rebound was notably more pronounced, with N levels reaching 2- and 4-fold increases over the control condition (Figure 5A). S gene expression followed the same trend, with an initial decrease at 6 hpi for both mimics (*p* < 0.001), followed at 24 hpi by a strong upregulation to about 2- and 4-fold increases above control, in contrast to the modest S downregulation observed for the Beta variant at this time point. As indicated previously, RNA levels signify genomic abundance without discriminating between viral viability. Therefore, viral RNA quantification and back titration experiments revealed productive infection in all conditions (Figure 5B,C). Unlike the Beta variant, for which miR-15b mimic reduced infectious progeny, neither miR-15b nor miR-29a mimics inhibited Omicron BA.1 replication. At 24 hpi, miR-29a-transfected cells yielded an approximately 1-log_10_ increase in infectious titer relative to control, indicating a paradoxical proviral effect, while miR-15b-transfected cells showed a modest increase (Figure 5C). This contrasting outcome relative to Beta may reflect variant-specific differences in spike processing and entry routes in Calu-3 cells, including altered Furin cleavage efficiency and TMPRSS2 usage.

#### 2.4.2. miRNA Inhibitor Experiments

**Figure 6:**
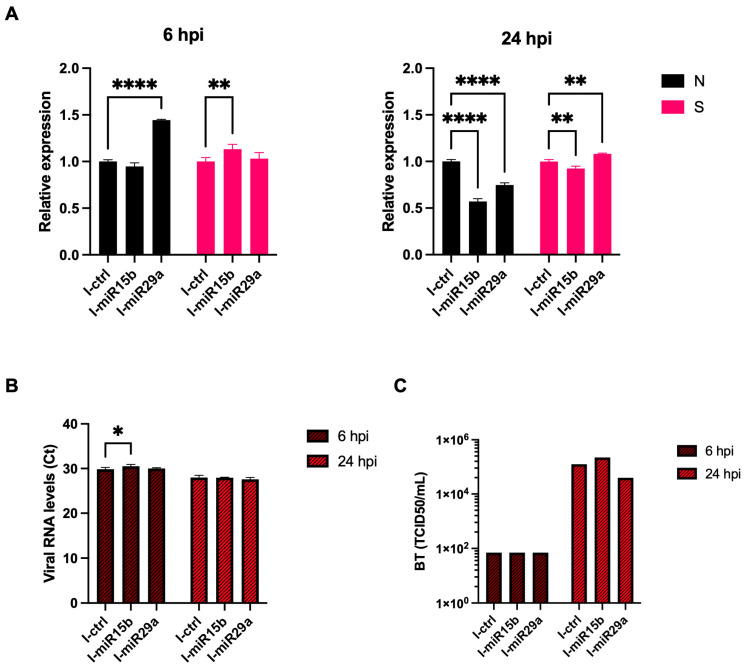
miRNA inhibitor effects in Omicron BA.1-infected Calu-3 cells. (**A**) N and S gene expression at 6 and 24 hpi in Calu-3 cells infected with Omicron BA.1 (0.1 MOI) and transfected with the control inhibitor (I-Ctrl, 50 nM), anti-miR-29a inhibitor (I-miR-29a, 50 nM), or anti-miR-15b inhibitor (I-miR-15b, 50 nM). Data are from RT-qPCR and expressed as fold change relative to the control inhibitor at each time point. (**B**) Viral RNA levels in cell culture supernatants at 6 and 24 hpi, quantified by RT-qPCR (Ct values for N RNA). (**C**) Infectious viral titers at 24 hpi in supernatants, determined as TCID_50_/mL using back titration on VeroE6/TMPRSS2 cells. Replicate structure is as in Figure 3. Data are from an independent experimental session from Figure 5. Bars represent mean ± SD. * *p* < 0.05; ** *p* < 0.01; **** *p* < 0.0001.

During Omicron BA.1 infection, inhibition of endogenous miR-15b or miR-29a in Calu-3 cells produced variant- and time-dependent effects on viral gene expression and infectivity. At 6 hpi, N transcript levels were significantly increased only upon miR-29a inhibition (*p* < 0.0001), while miR-15b inhibition left N expression comparable to the inhibitor control (Figure 6A). S mRNA showed the opposite pattern, with a modest but significant increase in the miR-15b inhibitor condition (*p* < 0.01), whereas miR-29a inhibition did not alter S levels relative to control. By 24 hpi, both inhibitors reduced N mRNA compared with control (*p* < 0.0001), while S transcript levels were slightly but significantly higher in the miR-29a inhibitor condition (*p* < 0.01) and marginally reduced with miR-15b inhibition (*p* < 0.01). Extracellular viral RNA in supernatants confirmed productive Omicron BA.1 replication under all conditions, with a small but statistically significant increase in Ct at 6 hpi in the presence of the miR-15b inhibitor (~0.5 Ct shift, corresponding to an approximately 1.4-fold reduction in viral RNA; *p* < 0.05) and comparable levels across all groups at 24 hpi (Figure 6B). BT assays showed that infectious Omicron progeny were comparable across all conditions at 6 hpi. At 24 hpi, miR-15b inhibition was associated with a slight increase in titer relative to control, whereas miR-29a inhibition yielded a modest reduction (~0.5 log_10_ below control) (Figure 6C).

### 2.5. Cellular Targets Modulation

**Figure 7:**
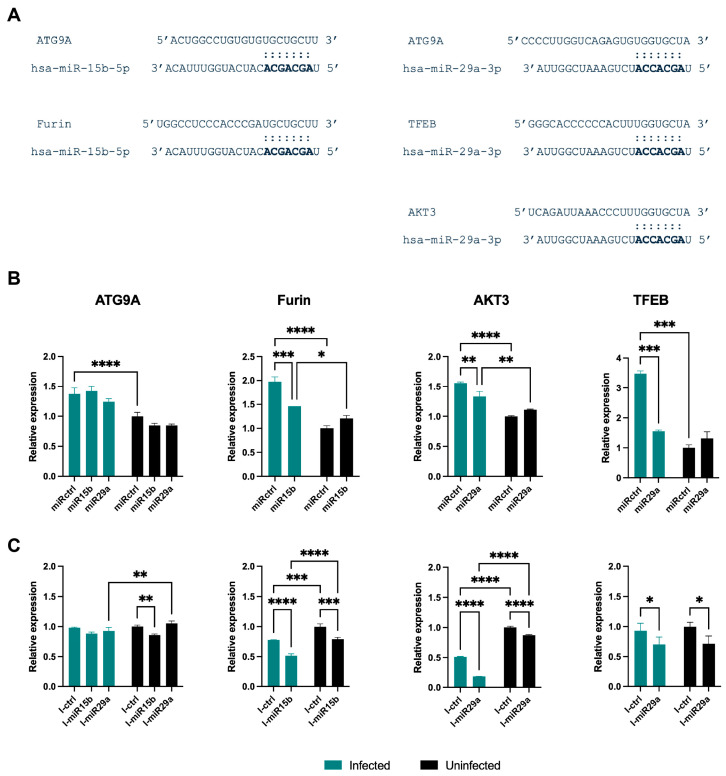
(**A**) miRNA–mRNA pairings as predicted by TargetScan and miRDB, or validated in the case of miR-29a vs. AKT3 [22], or vs. ATG9A [23]; seed sequences (2–8 nucleotides of miRNA 5′ region) are essential for target recognition and are shown in bold; they perfectly match the targets. (**B**) Cellular target modulation in Beta-infected cells. Expression of predicted cellular targets ATG9A, Furin, AKT3, and TFEB in SARS-CoV-2 Beta-infected Calu-3 cells under six transfection conditions: control miRNA, miR-29a mimic, miR-15b mimic, and (**C**) control inhibitor (I-ctrl), anti-miR-29a inhibitor (I-miR-29a), anti-miR-15b inhibitor (I-miR-15b). mRNA levels were quantified by RT-qPCR, normalized to GAPDH, and expressed as fold change relative to uninfected transfected cells (set to 1.0). One biological well per condition was assayed in triplicate by RT-qPCR (*n* = 3 technical replicates). Data are from a single experimental session conducted under BSL-3 containment. Bars represent mean ± SD. * *p* < 0.05; ** *p* < 0.01; *** *p* < 0.001; **** *p* < 0.0001.

As the antiviral effect on viral transcripts appeared to extend beyond direct viral RNA targeting, it can be hypothesized that some other miRNA cellular targets may be modulated and may contribute to the host–virus interaction. Thus, an inspection of the literature and predictive bioinformatic tools led to the evaluation of the expression of predicted and/or validated cellular targets Autophagy Related 9A (ATG9A), Furin, AKT Serine/Threonine Kinase 3 (also known as Protein Kinase B Gamma, PKBγ) (AKT3), and Transcription Factor EB (TFEB) in both infected and uninfected Calu-3 cells under mimic and inhibitor conditions [11,22,23]. Infection alone significantly altered the expression of these genes compared with uninfected cells, indicating that Beta infection per se remodels autophagy- and trafficking-related pathways (control miRNA-infected vs. control miRNA-uninfected: *p* < 0.0001 for ATG9A, Furin, and AKT3; *p* < 0.001 for TFEB, Figure 7A). Overexpression of miR-15b significantly reduced Furin mRNA levels in infected cells compared to the infected control miRNA (*p* < 0.001), and this reduction was partially maintained in uninfected cells (*p* < 0.05). Similarly, miR-29a mimic transfection significantly downregulated AKT3 (*p* < 0.01) and TFEB (*p* < 0.001) expression in infected cells relative to the infected control, and attenuated ATG9A levels, with the difference reaching significance when comparing infected control miRNA to uninfected miR-29a-transfected cells (*p* < 0.0001). Notably, miRNA-mediated effects on these cellular targets were more pronounced under infection conditions than in uninfected cells, consistent with the well-established principle that individual miRNAs typically exert modest fold-changes on endogenous targets at steady state [24,25], and that such regulatory effects are frequently amplified or unmasked under conditions of cellular stress or perturbation, when infection-induced transcriptomic remodeling alters mRNA stability, transcriptional output, and the competing endogenous RNA landscape of host transcripts [26,27].

Loss-of-function experiments using miRNA inhibitors provided complementary evidence (Figure 7B). Inhibition of miR-15b in infected cells resulted in a significant reduction in Furin expression relative to the control inhibitor in infected cells (*p* < 0.0001) and the uninfected anti-miR-15b inhibitor group (*p* < 0.001). Notably, this directionally mirrors the effect observed with miR-15b overexpression in Panel A, suggesting that the relationship between miR-15b and Furin regulation may involve indirect or context-dependent mechanisms beyond direct target repression. Strikingly, miR-29a inhibition in infected cells led to a marked further reduction in AKT3 expression relative to both the infected control inhibitor (*p* < 0.0001) and the uninfected anti-miR-29a inhibitor (*p* < 0.0001), indicating that basal miR-29a activity is required to maintain AKT3 expression in the context of infection. Modest but significant reductions in TFEB expression were also observed upon miR-29a inhibition in both infected and uninfected conditions (*p* < 0.05). In contrast, ATG9A expression was only marginally affected by inhibitor transfection, with small but significant differences detected between the infected and uninfected anti-miR-15b inhibitor or anti-miR-29a inhibitor groups (*p* < 0.01).

Collectively, these data demonstrate that infection induces the transcriptional upregulation of ATG9A, Furin, AKT3, and TFEB, and that miR-15b and miR-29a exert regulatory control over specific subsets of these genes, with miR-15b primarily targeting Furin and miR-29a modulating AKT3 and TFEB expression in an infection-dependent manner.

## 3. Discussion

This study systematically evaluated the functional role of miR-29a and miR-15b in SARS-CoV-2 Beta and Omicron BA.1 infection in lung epithelial Calu-3 cells. Both miRNAs directly bound the Spike (S) transcript and reduced its expression in plasmid-based overexpression systems (Figure 1B), a controlled setting designed to demonstrate direct molecular targeting capacity independently of the complex dynamics occurring during productive infection [4,5]. Computational prediction further identified conserved binding sites across the SARS-CoV-2 genome, suggesting that both miRNAs could suppress multiple viral transcripts across variants of concern (VOCs), establishing a baseline capacity for direct viral targeting. Although SARS-CoV-2 replication occurs within double-membrane vesicles (DMVs), these compartments are not fully sealed: cryo-electron tomography has revealed approximately 6 nm pores connecting the DMV lumen to the cytoplasm [28], and a substantial fraction of viral subgenomic RNAs is present in the cytoplasm for ribosomal translation, where RNA-induced silencing complex (RISC)-mediated silencing operates. This is consistent with precedent from other positive-sense single-stranded RNA (+ssRNA) viruses, including hepatitis C virus (HCV), where miRNA-mediated regulation occurs in the cytoplasm independently of membrane-enclosed replication compartments [29,30]. Endogenous miRNA levels were differentially modulated following infection: the Beta variant induced significant early upregulation followed by a later decrease, suggesting a dynamic, variant-specific interplay between viral replication and host miRNA regulation, whereas Omicron BA.1 infection did not significantly alter their expression. The absence of significant changes following Omicron infection does not contradict the functional effects observed upon mimic transfection; rather, it suggests that endogenous miRNA abundance may be insufficient to exert detectable antiviral pressure at physiological concentrations, analogous to what has been described during influenza A virus (IAV) infection, where endogenous miRNA levels are refractory to viral suppression yet exogenous mimic delivery produces significant antiviral effects [31]. These observations prompted evaluation of miRNA activity in a more complex system, in which cells were transfected with mimics or inhibitors 12 h prior to infection, followed by gene expression analysis and back-titration. The infection experiments revealed a more complex regulatory logic: miR-15b overexpression suppressed infectious particle production during Beta infection despite minimal effects on total extracellular viral RNA, whereas miR-29a paradoxically enhanced Omicron BA.1 infectivity at 24 h post-infection (hpi), despite early reductions in S transcripts. The choice to assess target modulation at the mRNA level is supported by evidence that mRNA destabilization accounts for the majority of miRNA-mediated silencing in mammalian cells, with translational inhibition contributing only approximately 10 to 25% of the total effect [32,33,34], and that mRNA-level repression is the earliest detectable molecular event following miRNA activity [24,25,35]. This discordance between transcript-level effects and infectivity phenotypes points to a critical role for post-transcriptional regulation of virion assembly and maturation beyond direct viral RNA targeting.

The parallel analysis of infection-regulated cellular targets provides a possible mechanistic basis for this finding. miR-15b overexpression reduced Furin mRNA levels relative to infected controls. Furin is a proprotein convertase required for S1/S2 cleavage and virion infectivity, and Furin-mediated processing is a prerequisite for efficient Beta variant entry in Calu-3 cells [36]. Notably, both overexpression and inhibition of miR-15b converged on reduced Furin mRNA, an outcome inconsistent with canonical direct target repression and suggestive of indirect regulatory mechanisms or compensatory feedback loops. A discrepancy between mimic and inhibitor experimental sessions, in which infected control cells showed elevated target gene expression relative to uninfected counterparts in mimic experiments but the opposite trend in inhibitor experiments, likely reflects inter-experiment variability and cautions against a strictly linear interpretation of miR-15b-mediated Furin regulation.

The regulatory impact of miR-29a appears to operate through a distinct axis involving autophagy and lysosomal biogenesis. Transcription factor EB (TFEB) expression was markedly elevated during infection, indicating robust activation of these pathways, and this upregulation was predominantly proviral. miR-29a overexpression reduced TFEB levels toward those observed in uninfected cells, suggesting that this miRNA can counteract virus-induced activation of this transcription factor. AKT serine/threonine kinase 3 (AKT3) displayed a similar, albeit less pronounced, pattern. These findings align with the established role of miR-29a in controlling the AKT–mTOR–TFEB axis [22] and suggest that this miRNA may limit viral exploitation of autophagy–lysosomal processes for replication and maturation [37,38,39]. While some observed changes in cellular target expression are modest, this is consistent with miRNA-mediated fine-tuning, where individual miRNAs typically repress endogenous targets by 1.2- to 2-fold at the mRNA level [24,25]. The coordinated targeting of multiple nodes, Furin in the processing pathway and TFEB and AKT3 in the autophagy–lysosomal axis, can nonetheless produce cumulative phenotypic outcomes, as reflected in the back-titration results [40]. Direct verification of this model would require assessment of Spike cleavage status on released virions, for example, through Western blot quantification of S1 and S2 subunits in concentrated supernatants, representing an important direction for future mechanistic studies.

The divergent miRNA effects between Beta and Omicron BA.1 likely reflect variant-specific differences in protease usage. Omicron BA.1 carries mutations in the Furin cleavage site and shows markedly reduced dependence on TMPRSS2-mediated entry compared with Beta, relying instead on cathepsin-mediated or alternative protease pathways [41,42,43]. Consequently, miR-15b-associated Furin reduction exerts less restrictive pressure on Omicron replication, and the concurrent elevation of TFEB-mediated autophagy becomes predominantly proviral, explaining the paradoxical enhancement of infectious particle production at late time points. This variant-dependent switching from antiviral to proviral phenotypes underscores a key principle: the net outcome of miRNA regulation is determined not by the miRNA itself but by the cellular and viral context, including variant-specific protease requirements and the balance between degradative and replicative autophagy.

This principle is further supported by the broader involvement of miR-29a and miR-15b in other viral infections. miR-29a inhibits IAV replication by targeting Frizzled 5 [44], and IAV infection downregulates miR-29a in A549 cells and mouse lungs, paralleling the temporal decrease observed here during Beta infection and suggesting a conserved viral strategy to counteract miR-29-mediated restriction. miR-29c modulates IAV-induced innate immune responses through A20 mRNA stabilization [45], and miR-29a targets HIV-1 Nef to facilitate P-body-mediated silencing [46,47]. Conversely, miR-15b promotes hepatitis B virus (HBV) replication by targeting hepatocyte nuclear factor 1α [48], contrasting with its antiviral effect in our Beta model and further reinforcing the context-dependency of miRNA function.

These findings must be interpreted within several methodological constraints. The use of a single cell line (Calu-3) limits generalizability to other tissues and cell types with distinct autophagy capacity, protease availability, and miRNA expression profiles [49]. The transient transfection of mimics and inhibitors represents a functional perturbation rather than physiological modulation and may not fully recapitulate infection-induced miRNA dynamics in vivo. The effect of inhibitors, ranging from no detectable effect to effects paralleling the mimics rather than opposing them (e.g., Figure 3C vs. Figure 4C, Figure 5C vs. Figure 6C), likely reflects these limitations and remains to be fully explained as understanding of miRNA–virus interactions evolves. BSL-3 constraints limited the number of biological replicates per session; however, mimic and inhibitor experiments were performed in independent sessions with independent cell preparations, and concordance across multiple readouts (intracellular transcripts, extracellular viral RNA, and infectious titers) within each session supports reliability. GAPDH was used as a reference gene consistent with its established use in SARS-CoV-2 Calu-3 studies [50,51,52], and key comparisons were performed within matched infection conditions to minimize normalization bias. The focus on two variants does not capture the full spectrum of variant-dependent regulation, and TCID_50_ determinations represent single measurements precluding formal statistical analysis; these values should be interpreted as descriptive observations concordant with parallel molecular readouts.

Despite these limitations, the findings identify miR-15b and miR-29a, together with their host targets Furin and TFEB, as potential modulatory nodes in the SARS-CoV-2 infection cycle. The experimental design employs a preventive model and does not directly support therapeutic applications in established infections. Future work should prioritize protein-level validation of Furin and TFEB modulation; post-infection delivery of mimics and inhibitors; variant-specific evaluation given the context-dependent directionality of effects; dissection of the indirect mechanisms linking miR-15b to Furin regulation before considering combination approaches with Furin inhibitors [53]; and studies in primary respiratory epithelia, macrophages, and physiologically relevant 3D and in vivo models [54]. Taken together, our results establish a molecular baseline justifying further investigation into miRNA-mediated modulation of host–virus interactions, while highlighting the complexity and context-dependency that must be addressed before these findings can inform therapeutic strategies.

## 4. Materials and Methods

### 4.1. Cell Lines

Human lung epithelial Calu-3 cells (ATCC, Manassas, VA, USA; cat. HTB-55; RRID: CVCL_0609) and A549 cells (ATCC; cat. CCL-185; RRID: CVCL_0023) were cultured in Dulbecco’s Modified Eagle’s Medium (DMEM), supplemented with 10% fetal bovine serum (FBS), 2 mM L-glutamine, and penicillin/streptomycin, at 37 °C in a humidified 5% CO_2_ incubator. HEK-293T cells (ATCC; cat. CRL-3216; RRID: CVCL_0063), Vero E6 cells (Vero C1008, clone E6; ATCC; cat. CRL-1586; RRID: CVCL_0574), and VeroE6/TMPRSS2 cells (NIBSC, Potters Bar, UK; cat. 100978; RRID: CVCL_YQ49) were maintained under the same conditions and used for viral stock preparation and titration. For VeroE6/TMPRSS2 cells, 1 mg/mL Geneticin (G418) was included in the culture medium to preserve TMPRSS2 expression. All cell lines were obtained from authenticated repositories and were routinely screened for mycoplasma contamination using a commercial assay (Lonza, Basel, Switzerland; cat. LT07-218).

### 4.2. Viruses and Infection Conditions

SARS-CoV-2 Beta (B.1.351, GISAID accession ID: EPI_ISL_1599180) and Omicron BA.1 (GISAID accession ID EPI_ISL_12188061) variants were propagated on VeroE6/TMPRSS2 cells. Viral stocks were quantified by Endpoint Dilution Assay on Vero E6 cells. Cells (4 × 10^5^ cells/mL) were seeded in 96-well plates and infected at 95% confluency with serial 10-fold viral dilutions. Following 1 h adsorption at 37 °C, the inoculum was removed, cells were washed with PBS, and complete medium was added. The cytopathic effect was assessed at 72 h, and viral titers (TCID_50_/mL) were calculated using the Reed–Muench method. Infections were performed at a multiplicity of infection (MOI) of 0.1 in serum-free medium for 1 h at 37 °C, after which the medium was replaced with DMEM containing 2% FBS. This MOI and infection protocol were consistent across all experiments to ensure comparability.

### 4.3. miRNA Mimics and Inhibitors

miR-29a-3p (hsa-miR-29a-3p) and miR-15b-5p (hsa-miR-15b-5p) mimics (miRIDIAN microRNA) and inhibitors (miRIDIAN microRNA Hairpin inhibitor), as well as their corresponding negative control molecules (miRIDIAN microRNA Mimic Negative control 1 and miRIDIAN microRNA hairpin Inhibitor Negative Control 1), were obtained from commercial suppliers (Dharmacon, Lafayette, CO, USA). Transfections were performed at a final concentration of 50 nM per molecule using Lipofectamine 2000 transfection reagent (Invitrogen, ThermoFisher, 168 Third Avenue, Waltham, MA, USA), according to manufacturer instructions, 6 h before infection (for infection experiments) or 24 h before harvesting (for uninfected cell experiments). The increased expression of miR-29a and miR-15b in comparison to the control after mimic transfection experiments ranged from 150- to 200-fold at the time of the assays.

### 4.4. Plasmid Constructs and S Expression Assays

Mammalian expression plasmid encoding the SARS-CoV-2 S protein from Wuhan-Hu-1 (pUNO1-SARS2-S, here called pSpike, InvivoGen, San Diego, CA, USA) was used to transfect cells. A549 and Calu-3 cells were co-transfected in 24-well plates with pSpike (0.5 μg per well) and either miR-29a mimic, miR-15b mimic, or the negative control miRNA (50 nM each) using 3 μL of Lipofectamine 2000 (Invitrogen, ThermoFisher) for 1 μg of nucleic acids, as described by the manufacturer. At 48 h post-transfection, cells were lysed and total RNA was extracted for RT-qPCR analysis of S gene expression, normalized to GAPDH. For HEK-293T cells, miR-29a mimic, miR-15b mimic, or control miRNA (50 nM) were first transfected using Lipofectamine 2000. After 24 h, cells were transfected again with plasmids encoding S protein (100 ng per well in 96-well plates). At 48 h after plasmid transfection, cells were lysed, and total RNA was extracted for RT-qPCR analysis of gene expression, normalized to GAPDH.

### 4.5. RT-qPCR Quantification of Viral and Cellular Genes

Total RNA from infected and control cells was extracted using the miRNeasy Mini Kit (Qiagen) according to the manufacturer’s protocol, whereas viral RNA in cell culture supernatants was purified with the QIAamp Viral RNA Mini Kit (QIAGEN). The RNA concentration was determined spectrophotometrically (NanoDrop 2000c, ThermoScientific, Waltham, MA, USA). cDNA preparations were obtained using the SuperScript™ First-Strand Synthesis System for RT-PCR (Thermo Fisher Scientific, Waltham, MA, USA). Real-time reverse transcriptase-PCR (Real-time RT-PCR) was performed on a ABI-PRISM 7900HT Fast Real-Time instrument (Applied Biosystems, Foster City, CA, USA) using SYBR Green chemistry and optical-grade 96-well plates with 20 μL reaction volumes run in duplicate with the following primers: N, 5′-TTACAAACATTGGCCGCAAA-3′ and 5′-GCGCGACATTCCGAA-3′; S, 5′-TCAACTCAGGACTTGTTCTTAC-3′ and 5′-TGGTAGGACAGGTTATCAAAC-3′; ATG9A, 5′-CCCCAGTACTGCCACCTTTA-3′ and 5′-ACAGCCTGACCTGCTCATCT-3′ [55]; Furin, 5′-ACAACTATGGGACGCTGACC-3′ and 5′-TGGACACAGCTCTTCTGGTG-3′ [56]; AKT3, 5′-TGTGGATTTACCTTATCCCCTCA-3′ and 5′-GTTTGGCTTTGGTCGTTCTGT-3′ [57]; TFEB, 5′-CCAGAAGCGAGAGCTCACAGAT-3′ and 5′-TGTGATTGTCTTTCTTCTGCCG-3′ [58]; and GAPDH (reference transcript), 5′-GAAGGTGAAGGTCGGAGTC-3′ and 5′-GAAGATGGTGATGGGATTT-3′. As SARS-CoV-2 infection can affect global host transcriptional output through NSP1-mediated host shutoff mechanisms [59,60,61], all comparative analyses of cellular target expression were performed within matched infection conditions (i.e., miRNA-transfected vs. control-transfected cells under the same infection status) to minimize potential normalization bias.

miR-29a-3p and miR-15b-5p levels were assessed via RT-qPCR using TaqMan-based miRNA detection (Applied Biosystems), with RNU6B serving as the endogenous normalizer. Assays were conducted according to the kit manufacturer’s guidelines. All reactions were performed in triplicate, and data were analyzed using the ΔΔCt method. Results were expressed as fold changes relative to control-transfected cells, uninfected cells (set to 1.0) or normalized to mock-infected controls. Statistical significance was assessed using Student’s *t*-test with a significance threshold of *p* < 0.05.

### 4.6. Back Titration and Measurement of Infectious Progeny

VeroE6/TMPRSS2 cells (4 × 10^5^ cells/mL) were distributed into 96-well plates and exposed to serial 10-fold dilutions of collected medium or cell lysates in triplicate. Following 1 h viral adsorption at 37 °C, cells were washed with PBS and supplied with fresh complete medium. At 72 h post-infection, cultures were examined for the presence of cytopathic effects, with viral titers determined using the Reed–Muench calculation (as previously described).

### 4.7. Statistical Analysis and Bioinformatic Tools

Data are presented as mean ± standard deviation (SD) of at least two independent biological replicates, each containing at least three technical replicates. All major trends were reproducible across independent experiments. Statistical significance was determined by two-tailed Student’s *t*-test; *p*-values < 0.05 were considered significant. Where applicable, two-way ANOVA was used to evaluate the effects of miRNA treatment and time point or variant. The MiRanda algorithm [62], RNAhybrid 2.2 and miRDB tools were used to predict binding sites on the SARS-CoV-2 genome [63]. For VOCs, sequence alignments were performed to assess conservation of predicted target sites using GISAID-derived sequences. Cellular targets (ATG9A, Furin, AKT3, TFEB) were selected based on published validations in other systems and in silico predictions from TargetScan and miRDB [22,23].

## 5. Conclusions

This study demonstrates that endogenous miR-29a and miR-15b, despite targeting the SARS-CoV-2 genomic and subgenomic RNAs and being involved in host pathways relevant to viral infection, do not exert straightforward antiviral effects in Beta- and BA.1 Omicron-infected lung epithelial cells. Instead, selective conditions, particularly miR-15b overexpression and inhibition of either miRNA, paradoxically enhanced infectious progeny production, underscoring the non-linear relationship between miRNA levels and infection outcome. These findings caution against oversimplified therapeutic designs based on miRNA delivery and instead advocate for mechanistic, context-aware approaches that integrate miRNA function with cell type, viral variant, and immune status. Future work dissecting the temporal dynamics of miRNA target modulation, coupled with primary cell and in vivo models, will be necessary to unlock the potential of miRNAs as antiviral agents.

## Data Availability

The datasets presented in this study can be found in online repositories. The names of the repository/repositories and accession number(s) can be found below: GISAID (https://gisaid.org/, accessed on 9 May 2026). The IDs of the uploaded data are: Beta 1.351 (GISAID accession ID: EPI_ISL_1599180) and Omicron BA.1 (GISAID accession ID: EPI_ISL_12188061).

## Supplementary Materials

The following supporting information can be downloaded at: https://www.mdpi.com/article/10.3390/ijms27135847/s1.

## Abbreviations

The following abbreviations are used in this manuscript:

A549	Human lung adenocarcinoma epithelial cell line
ACE2	Angiotensin-converting enzyme 2
AKT3	AKT serine/threonine kinase 3
ATG9A	Autophagy-related 9A
BA.1	Omicron BA.1 sub-lineage (B.1.1.529)
BSL-3	Biosafety Level 3
BT	Back titration
Calu-3	Human lung adenocarcinoma cell line
COVID-19	Coronavirus disease 2019
CPE	Cytopathic effect
Ct	Cycle threshold
DMEM	Dulbecco’s Modified Eagle’s Medium
EDA	Endpoint dilution assay
FBS	Fetal bovine serum
GAPDH	Glyceraldehyde-3-phosphate dehydrogenase
GISAID	Global Initiative on Sharing All Influenza Data
HBV	Hepatitis B virus
HEK-293T	Human embryonic kidney 293T cells
HIV-1	Human immunodeficiency virus type 1
hpi	Hours post-infection
hsa	*Homo sapiens*
IAV	Influenza A virus
I-Ctrl	Control inhibitor (scrambled anti-miRNA)
I-miR-15b	Anti-miR-15b hairpin inhibitor
I-miR-29a	Anti-miR-29a hairpin inhibitor
miR-15b	hsa-miR-15b-5p
miR-29a	hsa-miR-29a-3p
miRNA	MicroRNA
MOI	Multiplicity of infection
mTOR	Mechanistic target of rapamycin
N	Nucleocapsid protein/gene
NIBSC	National Institute for Biological Standards and Control
NSP1	Non-structural protein 1
ORF	Open reading frame
PBS	Phosphate-buffered saline
pSpike	Spike-expressing plasmid (pUNO1-SARS2-S)
RdRp	RNA-dependent RNA polymerase
RISC	RNA-induced silencing complex
RNU6B	RNA, U6 small nuclear B (reference small RNA)
RT-qPCR	Real-time quantitative reverse transcription PCR
S	Spike protein/gene
SARS-CoV-2	Severe acute respiratory syndrome coronavirus 2
SD	Standard deviation
TCID_50_	50% tissue culture infectious dose
TFEB	Transcription factor EB
TMPRSS2	Transmembrane serine protease 2
UTR	Untranslated region
Vero E6	African green monkey kidney epithelial cells (clone E6)
VOC	Variant of concern

## References

[1] Saini S., Saini A., Thakur C.J., Kumar V., Gupta R.D., Sharma J.K. (2020). Genome-Wide Computational Prediction of miRNAs in Severe Acute Respiratory Syndrome Coronavirus 2 (SARS-CoV-2) Revealed Target Genes Involved in Pulmonary Vasculature and Antiviral Innate Immunity. Mol. Biol. Res. Commun..

[2] Xu D.-Y., Zhou X., Ren Y.-J. (2025). RNAi-Based Antiviral Immunity. Yi Chuan Hered..

[3] Russo A., Potenza N. (2011). Antiviral Effects of Human microRNAs and Conservation of Their Target Sites. FEBS Lett..

[4] Fossat N., Lundsgaard E.A., Costa R., Rivera-Rangel L.R., Nielsen L., Mikkelsen L.S., Ramirez S., Bukh J., Scheel T.K.H. (2023). Identification of the Viral and Cellular microRNA Interactomes during SARS-CoV-2 Infection. Cell Rep..

[5] Siniscalchi C., Di Palo A., Russo A., Potenza N. (2021). Human MicroRNAs Interacting With SARS-CoV-2 RNA Sequences: Computational Analysis and Experimental Target Validation. Front. Genet..

[6] Kriegel A.J., Liu Y., Fang Y., Ding X., Liang M. (2012). The miR-29 Family: Genomics, Cell Biology, and Relevance to Renal and Cardiovascular Injury. Physiol. Genom..

[7] Pathania A.S., Chava H., Chaturvedi N.K., Chava S., Byrareddy S.N., Coulter D.W., Challagundla K.B. (2024). The miR-29 Family Facilitates the Activation of NK-Cell Immune Responses by Targeting the B7-H3 Immune Checkpoint in Neuroblastoma. Cell Death Dis..

[8] Dong J., Huth W.J., Marcel N., Zhang Z., Lin L.-L., Lu L.-F. (2023). miR-15/16 Clusters Restrict Effector Treg Cell Differentiation and Function. J. Exp. Med..

[9] Mahdavi S.Z.B., Jebelli A., Aghbash P.S., Baradaran B., Amini M., Oroojalian F., Pouladi N., Baghi H.B., de la Guardia M., Mokhtarzadeh A.A. (2025). A Comprehensive Overview on the Crosstalk between microRNAs and Viral Pathogenesis and Infection. Med. Res. Rev..

[10] Moens U. (2009). Silencing Viral MicroRNA as a Novel Antiviral Therapy?. BioMed Res. Int..

[11] Hardin L.T., Xiao N. (2022). miRNAs: The Key Regulator of COVID-19 Disease. Int. J. Cell Biol..

[12] Criscuolo E., Giuliani B., Ferrari D., Ferrarese R., Diotti R.A., Clementi M., Mancini N., Clementi N. (2022). Proper Selection of In Vitro Cell Model Affects the Characterization of the Neutralizing Antibody Response against SARS-CoV-2. Viruses.

[13] Criscuolo E., Giuliani B., Castelli M., Cavallaro M., Sisti S., Burioni R., Ferrari D., Mancini N., Locatelli M., Clementi N. (2024). Single Spike Mutation Differentiating XBB.1 and XBB.1.5 Enhances SARS-CoV-2 Cell-to-Cell Transmission and Facilitates Serum-Mediated Enhancement. Front. Immunol..

[14] Hoffmann M., Kleine-Weber H., Schroeder S., Krüger N., Herrler T., Erichsen S., Schiergens T.S., Herrler G., Wu N.-H., Nitsche A. (2020). SARS-CoV-2 Cell Entry Depends on ACE2 and TMPRSS2 and Is Blocked by a Clinically Proven Protease Inhibitor. Cell.

[15] Matsuyama S., Nao N., Shirato K., Kawase M., Saito S., Takayama I., Nagata N., Sekizuka T., Katoh H., Kato F. (2020). Enhanced Isolation of SARS-CoV-2 by TMPRSS2-Expressing Cells. Proc. Natl. Acad. Sci. USA.

[16] Kong Y.W., Cannell I.G., de Moor C.H., Hill K., Garside P.G., Hamilton T.L., Meijer H.A., Dobbyn H.C., Stoneley M., Spriggs K.A. (2008). The Mechanism of Micro-RNA-Mediated Translation Repression Is Determined by the Promoter of the Target Gene. Proc. Natl. Acad. Sci. USA.

[17] Pawlica P., Yario T.A., White S., Wang J., Moss W.N., Hui P., Vinetz J.M., Steitz J.A. (2021). SARS-CoV-2 Expresses a microRNA-like Small RNA Able to Selectively Repress Host Genes. Proc. Natl. Acad. Sci. USA.

[18] Farr R.J., Rootes C.L., Rowntree L.C., Nguyen T.H.O., Hensen L., Kedzierski L., Cheng A.C., Kedzierska K., Au G.G., Marsh G.A. (2021). Altered microRNA Expression in COVID-19 Patients Enables Identification of SARS-CoV-2 Infection. PLoS Pathog..

[19] Desai N., Neyaz A., Szabolcs A., Shih A.R., Chen J.H., Thapar V., Nieman L.T., Solovyov A., Mehta A., Lieb D.J. (2020). Temporal and Spatial Heterogeneity of Host Response to SARS-CoV-2 Pulmonary Infection. Nat. Commun..

[20] Dagotto G., Mercado N.B., Martinez D.R., Hou Y.J., Nkolola J.P., Carnahan R.H., Crowe J.E., Baric R.S., Barouch D.H. (2021). Comparison of Subgenomic and Total RNA in SARS-CoV-2-Challenged Rhesus Macaques. J. Virol..

[21] Cheng Y.-H., Chen C.-H., Liu P.-C., Chen W.-T., Hsu C.-J., Chen C.-C., Sun J.-R. (2025). Reverse Transcription-Quantitative PCR Assays for Detecting SARS-CoV-2 Using Subgenomic RNA Load. Heliyon.

[22] Li P., Hao X., Liu J., Zhang Q., Liang Z., Li X., Liu H. (2023). miR-29a-3p Regulates Autophagy by Targeting Akt3-Mediated mTOR in SiO2-Induced Lung Fibrosis. Int. J. Mol. Sci..

[23] Xu Y., Yang J., Li F., Lian G., Ouyang M. (2018). MiR-29a Inhibited Intestinal Epithelial Cells Autophagy Partly by Decreasing ATG9A in Ulcerative Colitis. Anti-Cancer Drugs.

[24] Baek D., Villén J., Shin C., Camargo F.D., Gygi S.P., Bartel D.P. (2008). The Impact of microRNAs on Protein Output. Nature.

[25] Selbach M., Schwanhäusser B., Thierfelder N., Fang Z., Khanin R., Rajewsky N. (2008). Widespread Changes in Protein Synthesis Induced by microRNAs. Nature.

[26] Leung A.K.L., Sharp P.A. (2010). MicroRNA Functions in Stress Responses. Mol. Cell.

[27] Mendell J.T., Olson E.N. (2012). MicroRNAs in Stress Signaling and Human Disease. Cell.

[28] Wolff G., Limpens R.W.A.L., Zevenhoven-Dobbe J.C., Laugks U., Zheng S., de Jong A.W.M., Koning R.I., Agard D.A., Grünewald K., Koster A.J. (2020). A Molecular Pore Spans the Double Membrane of the Coronavirus Replication Organelle. Science.

[29] Jopling C.L., Yi M., Lancaster A.M., Lemon S.M., Sarnow P. (2005). Modulation of Hepatitis C Virus RNA Abundance by a Liver-Specific MicroRNA. Science.

[30] Thibault P.A., Huys A., Dhillon P., Wilson J.A. (2013). MicroRNA-122-Dependent and -Independent Replication of Hepatitis C Virus in Hep3B Human Hepatoma Cells. Virology.

[31] Peng S., Wang J., Wei S., Li C., Zhou K., Hu J., Ye X., Yan J., Liu W., Gao G.F. (2018). Endogenous Cellular MicroRNAs Mediate Antiviral Defense against Influenza A Virus. Mol. Ther. Nucleic Acids.

[32] Guo H., Ingolia N.T., Weissman J.S., Bartel D.P. (2010). Mammalian microRNAs Predominantly Act to Decrease Target mRNA Levels. Nature.

[33] Eichhorn S.W., Guo H., McGeary S.E., Rodriguez-Mias R.A., Shin C., Baek D., Hsu S., Ghoshal K., Villén J., Bartel D.P. (2014). mRNA Destabilization Is the Dominant Effect of Mammalian MicroRNAs by the Time Substantial Repression Ensues. Mol. Cell.

[34] Diener C., Keller A., Meese E. (2023). The miRNA–Target Interactions: An Underestimated Intricacy. Nucleic Acids Res..

[35] Jovanovic M., Rooney M.S., Mertins P., Przybylski D., Chevrier N., Satija R., Rodriguez E.H., Fields A.P., Schwartz S., Raychowdhury R. (2015). Dynamic Profiling of the Protein Life Cycle in Response to Pathogens. Science.

[36] Kumar S., Delipan R., Sharma C., Jadoun J., Kanjo K., Singh R., Rajmani R., Deshpande S., Pandey R., Thakur K.G. (2025). Spike Conformational and Glycan Heterogeneity Associated with Furin Cleavage Causes Incomplete Neutralization of SARS-CoV-2. Nat. Commun..

[37] Koči J., Novotová M., Sláviková M., Klempa B., Zahradník I. (2022). SARS-CoV-2 Exploits Non-Canonical Autophagic Processes to Replicate, Mature, and Egress the Infected Vero E6 Cells. Pathogens.

[38] Zhou H., Hu Z., Castro-Gonzalez S. (2023). Bidirectional Interplay between SARS-CoV-2 and Autophagy. mBio.

[39] Gabig-Cimińska M. (2025). Dysregulated TFEB–Autophagy-Lysosome Pathway Links Acute COVID-19 Immunopathology to Long COVID Sequelae. Front. Immunol..

[40] Ebert M.S., Sharp P.A. (2012). Roles for MicroRNAs in Conferring Robustness to Biological Processes. Cell.

[41] Cheng M.T.K., Altaf M., Castin J., Reuschl A.-K., Sievers B.L., Kamelian K., Mesner D., Morse R.B., Abdullahi A., Meng B. (2025). Signatures of Omicron-like Adaptation in Early SARS-CoV-2 Variants and Chronic Infection. Cell Rep..

[42] Papa G., Mallery D.L., Albecka A., Welch L.G., Cattin-Ortolá J., Luptak J., Paul D., McMahon H.T., Goodfellow I.G., Carter A. (2021). Furin Cleavage of SARS-CoV-2 Spike Promotes but Is Not Essential for Infection and Cell-Cell Fusion. PLoS Pathog..

[43] Johnson B.A., Xie X., Bailey A.L., Kalveram B., Lokugamage K.G., Muruato A., Zou J., Zhang X., Juelich T., Smith J.K. (2021). Loss of Furin Cleavage Site Attenuates SARS-CoV-2 Pathogenesis. Nature.

[44] Yang X., Liang Y., Bamunuarachchi G., Xu Y., Vaddadi K., Pushparaj S., Xu D., Zhu Z., Blaha R., Huang C. (2021). miR-29a Is a Negative Regulator of Influenza Virus Infection through Targeting of the Frizzled 5 Receptor. Arch. Virol..

[45] Zhang X., Dong C., Sun X., Li Z., Zhang M., Guan Z., Duan M. (2014). Induction of the Cellular miR-29c by Influenza Virus Inhibits the Innate Immune Response through Protection of A20 mRNA. Biochem. Biophys. Res. Commun..

[46] Petrova V., Chitteni-Pattu S., Drees J.C., Inman R.B., Cox M.M. (2009). An SOS Inhibitor That Binds to Free RecA Protein: The PsiB Protein. Mol. Cell.

[47] Yao X.-C., Wu J.-J., Yuan S.-T., Yuan F.-L. (2025). Recent Insights and Perspectives into the Role of the miRNA-29 Family in Innate Immunity (Review). Int. J. Mol. Med..

[48] Dai X., Zhang W., Zhang H., Sun S., Yu H., Guo Y., Kou Z., Zhao G., Du L., Jiang S. (2014). Modulation of HBV Replication by microRNA-15b through Targeting Hepatocyte Nuclear Factor 1α. Nucleic Acids Res..

[49] Bestle D., Heindl M.R., Limburg H., Van Lam Van T., Pilgram O., Moulton H., Stein D.A., Hardes K., Eickmann M., Dolnik O. (2020). TMPRSS2 and Furin Are Both Essential for Proteolytic Activation of SARS-CoV-2 in Human Airway Cells. Life Sci. Alliance.

[50] Sun C., Xu F., Pu Z., Zhu H.-P., Li Y., Lu H.-J., Wu B.-B., Sun Y.-S., Yao P.-P., Jiang J.-M. (2025). MicroRNA-Mediated Regulation of the Immune Response in Calu-3 Cells Infected with a SARS-CoV-2 E Gene Variant. Front. Microbiol..

[51] Staroverov V., Galatenko A., Knyazev E., Tonevitsky A. (2024). Mathematical Model Explains Differences in Omicron and Delta SARS-CoV-2 Dynamics in Caco-2 and Calu-3 Cells. PeerJ.

[52] Corrêa I.A., de Souza M.R.M., da Silva G.P.D., Pimentel A.B.S.V.M., Calil P.T., Cunha M.S., Mariani D., de Moraes Brindeiro R., Costa S.M., da Costa Simas M.C. (2025). Replication Differences of SARS-CoV-2 Lineages May Arise from Unique RNA Replication Characteristics and Nucleocapsid Protein Expression. Front. Cell. Infect. Microbiol..

[53] Ivachtchenko A.V., Khvat A.V., Shkil D.O. (2024). Development and Prospects of Furin Inhibitors for Therapeutic Applications. Int. J. Mol. Sci..

[54] Meng B., Abdullahi A., Ferreira I.A.T.M., Goonawardane N., Saito A., Kimura I., Yamasoba D., Gerber P.P., Fatihi S., Rathore S. (2022). Altered TMPRSS2 Usage by SARS-CoV-2 Omicron Impacts Infectivity and Fusogenicity. Nature.

[55] Shi Y., Yang X., Xue X., Sun D., Cai P., Song Q., Zhang B., Qin L. (2020). HANR Enhances Autophagy-Associated Sorafenib Resistance Through miR-29b/ATG9A Axis in Hepatocellular Carcinoma. OncoTargets Ther..

[56] Luna C., Li G., Qiu J., Epstein D.L., Gonzalez P. (2011). MicroRNA-24 Regulates the Processing of Latent TGFβ1 during Cyclic Mechanical Stress in Human Trabecular Meshwork Cells through Direct Targeting of FURIN. J. Cell. Physiol..

[57] Meng Z., Shen W., Yu L., Tong F., He H., Hu Y., Wu W., Liu J. (2023). Bach1 Modulates AKT3 Transcription to Participate in Hyperglycaemia-Mediated EndMT in Vascular Endothelial Cells. Clin. Exp. Pharmacol. Physiol..

[58] Mubariz F., Saadin A., Lingenfelter N., Sarkar C., Banerjee A., Lipinski M.M., Awad O. (2023). Deregulation of mTORC1-TFEB Axis in Human iPSC Model of GBA1-Associated Parkinson’s Disease. Front. Neurosci..

[59] Thoms M., Buschauer R., Ameismeier M., Koepke L., Denk T., Hirschenberger M., Kratzat H., Hayn M., Mackens-Kiani T., Cheng J. (2020). Structural Basis for Translational Shutdown and Immune Evasion by the Nsp1 Protein of SARS-CoV-2. Science.

[60] Fisher T., Gluck A., Narayanan K., Kuroda M., Nachshon A., Hsu J.C., Halfmann P.J., Yahalom-Ronen Y., Tamir H., Finkel Y. (2022). Parsing the Role of NSP1 in SARS-CoV-2 Infection. Cell Rep..

[61] Ivanov K.I., Yang H., Sun R., Li C., Guo D. (2024). The Emerging Role of SARS-CoV-2 Nonstructural Protein 1 (Nsp1) in Epigenetic Regulation of Host Gene Expression. FEMS Microbiol. Rev..

[62] Enright A.J., John B., Gaul U., Tuschl T., Sander C., Marks D.S. (2003). MicroRNA Targets in Drosophila. Genome Biol..

[63] Abueg L.A.L., Afgan E., Allart O., Awan A.H., Bacon W.A., Baker D., Bassetti M., Batut B., Bernt M., Galaxy Community (2024). The Galaxy Platform for Accessible, Reproducible, and Collaborative Data Analyses: 2024 Update. Nucleic Acids Res..

