# Supplementary material for: miR-29a and miR-15b Modulate SARS-CoV-2 Beta and Omicron Infection in Human Lung Epithelial Cells
Source: Int J Mol Sci. 2026 Jun 29;27(13):5847. doi: 10.3390/ijms27135847 (PMC13362023; doi:10.3390/ijms27135847)
Supplement: Supplementary file 1 [file ijms-27-05847-s001.zip › ijms-4341772-supplementary.pdf]

Supplementary materials

miR-29a and miR-15b Modulate SARS-CoV-2 Beta and Omicron Infection in Human Lung Epithelial Cells

Table S1. Predicted miRNA binding sites for hsa-miR-15b-5p along the SARS-CoV-2 genome (B.1, Wuhan-Hu-1 reference, NC_045512.2), identified using miRanda algorithm (Version 3.3a).

Query	Target	Score	Energy	Position	Align. Length	Align. Rate	Precision
>hsa-miR-15b-5p	NC_045512.2	154.00	-16.70	8444 8465	17	70.59%	76.47%
>hsa-miR-15b-5p	NC_045512.2	153.00	-25.82	16842 16864	18	72.22%	94.44%
>hsa-miR-15b-5p	NC_045512.2	153.00	-15.74	28906 28928	18	66.67%	83.33%
>hsa-miR-15b-5p	NC_045512.2	152.00	-12.48	28921 28942	15	73.33%	80.00%
>hsa-miR-15b-5p	NC_045512.2	152.00	-14.95	29007 29030	18	72.22%	77.78%
>hsa-miR-15b-5p	NC_045512.2	150.00	-14.08	14549 14569	19	63.16%	78.95%
>hsa-miR-15b-5p	NC_045512.2	149.00	-17.13	14766 14788	14	78.57%	85.71%
>hsa-miR-15b-5p	NC_045512.2	149.00	-20.14	24079 24101	14	78.57%	85.71%
>hsa-miR-15b-5p	NC_045512.2	147.00	-20.42	8599 8621	16	75.00%	75.00%
>hsa-miR-15b-5p	NC_045512.2	146.00	-14.01	26710 26730	15	66.67%	86.67%
>hsa-miR-15b-5p	NC_045512.2	143.00	-13.98	3150 3170	16	68.75%	75.00%
>hsa-miR-15b-5p	NC_045512.2	143.00	-13.78	13077 13097	16	62.50%	81.25%
>hsa-miR-15b-5p	NC_045512.2	142.00	-19.01	22629 22652	20	65.00%	80.00%
>hsa-miR-15b-5p	NC_045512.2	142.00	-16.39	23374 23393	17	76.47%	88.24%
>hsa-miR-15b-5p	NC_045512.2	141.00	-17.71	19844 19864	10	90.00%	90.00%
>hsa-miR-15b-5p	NC_045512.2	140.00	-15.26	21412 21433	19	68.42%	84.21%
>hsa-miR-15b-5p	NC_045512.2	140.00	-12.72	24620 24641	7	100.00%	100.00%
>hsa-miR-15b-5p	NC_045512.2	140.00	-14.78	25669 25690	7	100.00%	100.00%
>hsa-miR-15b-5p	NC_045512.2	140.00	-15.65	27087 27108	18	66.67%	77.78%

Table S2. Predicted miRNA binding sites for hsa-miR-29a-3p along the SARS-CoV-2 genome (B.1, Wuhan-Hu-1 reference, NC_045512.2), identified using miRanda algorithm (Version 3.3a).

Query	Target	Score	Energy	Position	Align. Length	Align. Rate	Precision
>hsa-miR-29a-3p	NC_045512.2	164.00	-22.72	19465 19486	15	80.00%	93.33%
>hsa-miR-29a-3p	NC_045512.2	158.00	-22.23	11358 11381	21	71.43%	80.95%
>hsa-miR-29a-3p	NC_045512.2	154.00	-21.97	22326 22349	20	70.00%	75.00%
>hsa-miR-29a-3p	NC_045512.2	153.00	-19.61	21812 21833	20	80.00%	85.00%
>hsa-miR-29a-3p	NC_045512.2	152.00	-22.01	2060 2081	15	80.00%	93.33%
>hsa-miR-29a-3p	NC_045512.2	152.00	-20.51	28627 28649	17	76.47%	76.47%
>hsa-miR-29a-3p	NC_045512.2	151.00	-14.41	16268 16291	21	66.67%	71.43%
>hsa-miR-29a-3p	NC_045512.2	150.00	-21.55	4505 4526	17	64.71%	76.47%
>hsa-miR-29a-3p	NC_045512.2	146.00	-20.12	3714 3734	19	57.89%	78.95%
>hsa-miR-29a-3p	NC_045512.2	143.00	-16.59	5904 5927	21	61.90%	80.95%
>hsa-miR-29a-3p	NC_045512.2	143.00	-10.98	9556 9578	20	65.00%	80.00%
>hsa-miR-29a-3p	NC_045512.2	143.00	-13.16	14433 14455	16	75.00%	87.50%
>hsa-miR-29a-3p	NC_045512.2	143.00	-20.24	26044 26068	22	63.64%	77.27%

Query	Target	Score	Energy	Position	Align. Length	Align. Rate	Precision
>hsa-miR-29a-3p	NC_045512.2	142.00	-12.98	9362 9386	22	63.64%	68.18%
>hsa-miR-29a-3p	NC_045512.2	141.00	-14.80	7535 7556	20	70.00%	80.00%
>hsa-miR-29a-3p	NC_045512.2	140.00	-15.88	8315 8336	7	100.00%	100.00%
>hsa-miR-29a-3p	NC_045512.2	140.00	-13.82	20854 20875	7	100.00%	100.00%
